# Adult-onset autoimmune diabetes: comparative analysis of classical and latent presentation

**DOI:** 10.1186/s13098-020-00616-1

**Published:** 2020-12-03

**Authors:** Lúcia Fadiga, Joana Saraiva, Diana Catarino, João Frade, Miguel Melo, Isabel Paiva

**Affiliations:** 1 Endocrinology, Diabetes and Metabolism Department, Centro Hospitalar e Universitário de Coimbra, Coimbra, Portugal; 2 Faculty of Medicine, University of Coimbra, Coimbra, Portugal; 3 Clinical Pathology Department, Centro Hospitalar e Universitário de Coimbra, Coimbra, Portugal; 4 Instituto de Investigação e Inovação em Saúde (I3S)/Institute of Pathology and Immunology of the University of Porto (Ipatimup), Porto, Portugal

**Keywords:** Autoimmune disease, Diabetes complications, Diabetes mellitus, type 1, Latent autoimmune diabetes in adults, Insulin resistance

## Abstract

**Introduction:**

Adult-onset autoimmune diabetes (AID) has two different phenotypes: classic type 1 diabetes mellitus (T1DM), with insulin requirement just after diagnosis, and latent autoimmune diabetes in adults (LADA). The purpose of this study is to characterize patients with AID followed on a tertiary centre, comparing classic T1DM and LADA.

**Methods:**

We collected data from patients with diabetes and positive islet autoantibodies, aged 30 years old and over at diagnosis. Patients who started insulin in the first 6 months were classified as T1DM and patients with no insulin requirements in the first 6 months were classified as LADA. Data regarding clinical presentation, autoantibodies, A1C and C-peptide at diagnosis, pharmacologic treatment and complications were analysed.

**Results:**

We included 92 patients, 46 with classic T1DM and 46 with LADA. The percentage of females was 50% in T1DM group and 52.1% in LADA group. The median age at diagnosis was 38 years (IQR–15) for T1DM and 42 years (IQR–15) for LADA (*p *= 0.057). The median time between diagnosis of diabetes and diagnosis of autoimmune aetiology was 0 months in T1DM group and 60 months in LADA group (*p *< 0.001). The mean BMI at diagnosis was 24.1 kg/m^2^ in T1DM group and 26.1 kg/m^2^ in LADA group (*p *= 0.042). In T1DM group, 67.4% of the patients had more than one positive autoantibody, comparing to 41.3% of LADA patients (*p *= 0.012). There was no statistical difference in what concerns to title of GAD autoantibodies, A1C and C-peptide at diagnosis of autoimmune aetiology. The presence of symptoms at diagnosis was associated with T1DM group (*p *< 0.001). The median daily insulin dose was 40 IU for T1DM (0.58 IU/kg) and 33.5 IU for LADA (0.57 IU/kg), with no statistical difference. LADA patients were more often under non-insulin antidiabetic drugs (*p *= 0.001). At 10 years follow up, 21.1% of T1DM patients and 63.3% of LADA patients had microvascular complications (*p *= 0.004). Diabetic nephropathy was present in 23.5% of T1DM patients and 53.3% of LADA patients (*p *= 0.047). At the last evaluation, 55.6% of T1DM and 82.6% of LADA patients had metabolic syndrome and this difference was independent of diabetes duration.

**Conclusion:**

Patients with classic T1DM presented more often with symptoms, lower BMI and higher number of autoantibodies, which may be related to a more aggressive autoimmune process. Patients with LADA developed more frequently microvascular complications for the same disease duration, namely diabetic nephropathy, and had more often metabolic syndrome.

## Introduction

Adult-onset autoimmune diabetes (AID) is a complex and heterogeneous condition, whose main feature is the presence of serum diabetes-related autoantibodies [1, 2]. AID may present as a sudden onset of insulin deficiency, with symptoms and frequent ketosis, being the patient dependent on exogenous insulin just after the diagnosis-the so-called “classic type 1 diabetes mellitus” (T1DM). On the other hand, patients may present with slowly progressive insulin deficiency, variable levels of insulin resistance and often do not require insulin treatment for a considerable period after diagnosis-the so-called “latent autoimmune diabetes in adults” (LADA) [1, 3, 4]. According to the Immunology of Diabetes Society (IDS), LADA diagnosis is based on three criteria: a minimal age of 30 years at diabetes onset, the presence of circulating islet autoantibodies and lack of insulin requirement for at least 6 months after diagnosis [4].

Although LADA and T1DM share common genetic and immune characteristics, LADA patients also seem to match some features of type 2 diabetes (T2DM), like being often overweight/obese, physically inactive and having other criteria for the metabolic syndrome, which leads to insulin resistance [1, 3, 5]. In fact, both autoimmunity and insulin resistance play important roles in the pathogenesis of LADA and, depending on the predominant factor, the phenotype will be more T1DM-like or T2DM-like [1, 6].

The purpose of this study is to characterize patients with AID, comparing classic T1DM and LADA in what concerns to clinical presentation, metabolic control, pharmacologic treatment and diabetic complications.

## Methods

**Fig. 1:**
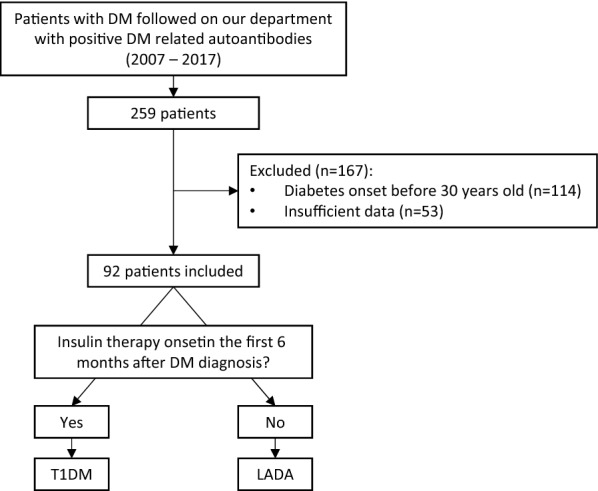
Representation of the study methodology

We conducted a retrospective cohort study at our tertiary care centre, including patients with diabetes mellitus (DM) and one or more positive diabetes-related autoantibodies—glutamic acid decarboxylase antibodies (GADA), islet cell antibodies (ICA), tyrosine phosphatase-like insulinoma antigen 2 antibodies (IA2) and endogenous insulin antibodies (IAA)—in blood samples analysed between the 1st January 2007 and the 30th June 2017 at our laboratory. ICA were analysed through indirect immunofluorescence (Mago 4^®^), a semi-quantitative method. GADA, IA2 and IAA were analysed through radioimmunoassay (Wallac Wizard 1470^®^ Automatic Gamma Counter), with reference values of 1.0 U/mL, 1.0 U/mL and 0.4 U/mL, respectively. We excluded patients with diabetes onset before 30 years of age (median age 25 years, IQR 5) and with relevant lack of clinical data, such as age of onset of diabetes or age of onset of insulin therapy (Fig. 1).

Patients who started insulin in the first 6 months after diabetes diagnosis were included in the T1DM group and patients with no insulin requirement in the first 6 months after diagnosis were included in the LADA group.

We reviewed patients’ clinical records and collected data regarding age, sex, weight, height, body mass index (BMI), date of diagnosis of diabetes, clinical presentation, haemoglobin A1c (A1C) and fasting C-peptide (C-pep) at diagnosis, date of autoantibodies measurement and autoantibodies levels.

All patients were evaluated at the last medical appointment in what concerns to autoimmune comorbidities, current pharmacologic treatment, hypertension, dyslipidaemia and metabolic syndrome. For the majority of autoimmune diseases, the screening was clinical or based in abnormal blood tests findings during follow-up (e.g. macrocytic anaemia for atrophic gastritis or abnormal liver function tests for autoimmune hepatitis); autoimmune thyroiditis was screened at least once a year through the analysis of thyroid antibodies.

Metabolic syndrome (MetS) was considered to be present if patients had 2 of 4 of the following criteria: hypertension, obesity, elevated triglycerides and low high-density lipoprotein cholesterol (or lipid-lowering drugs) [7]. Patients with at least 10 years of diabetes duration were assessed for diabetic complications at the mark of 10 years. Diabetic nephropathy was considered based on estimated glomerular filtration rate (GFR) and albuminuria, according to KDIGO 2012 guidelines [8]. The remaining complications were considered based on clinical records, including yearly evaluation at the Department of Ophthalmology.

Statistical analysis was performed using IBM SPSS Statistics v.23 for Windows. Normally distributed data are given as mean and standard deviation. Non-normally distributed data are given as median and range. Differences between groups were evaluated using independent-samples T-test and Mann–Whitney test and association between variables was assessed with Chi square test and Fisher’s exact test. A *p *< 0.05 value was considered significant.

## Results

We included 92 patients, 46 with T1DM and 46 with LADA. The descriptive characteristics of the patients, including anthropometric, clinical and biochemical data, as well as comparative analysis between both groups, are presented in Table 1.

**Table 1:** Clinical and biochemical characteristics of included patients

	T1DM (n = 46)	LADA (n = 46)	*p*
Females, n (%)	23 (50.0%)	24 (52.2%)	0.835
Age at diabetes diagnosis, median (IQR)	38 (15)	42 (15)	0.057
Months between diagnosis of diabetes and insulin therapy beginning, median (IQR)	0 (0.0)	48 (56.3)	< 0.001*
Months between diagnosis of diabetes and diagnosis of autoimmune aetiology, median (IQR)	0 (3.0)	60 (92.3)	< 0.001*
Diabetes duration at the last follow-up, years, median (IQR)	8.0 (7.5)	11.0 (10.5)	0.023*
At the diagnosis of diabetes			
Symptoms at presentation, n (%)	40 (87.0%)	21 (45.7%)	< 0.001**
Diabetic ketoacidosis, n	2	0	
Polyuric/polydipsic syndrome, n	38	21	
At the measurement of diabetes-related autoantibodies			
BMI (kg/m^2^), mean (± SD)	24.1 (± 3.8)	26.1 (± 5.2)	0.042***
A1C (%), mean (± SD)	10.3 (± 2.4)	9.5 (± 2.2)	0.113
C-peptide (ng/mL), median (IQR)	0.7 (0.6)	1.0 (1.4)	0.152
Number of positive antibodies, median (IQR)	2 (2.0)	1 (1.0)	0.013*
Patients with > 1 positive antibody, n (%)	31 (67.4%)	19 (41.3%)	0.012**
Positive GADA, n/total (%)	43/46 (93.5%)	39/46 (84.8%)	0.180
GADA title of patients with positive GADA (U/mL), median (IQR)	21.4 (94.1)	11.9 (60.4)	0.229
Positive ICA, n/total (%)	30/43 (69.8%)	23/38 (60.5%)	0.383
Positive IA2, n/total (%)	17/43 (39.5%)	10/45 (22.2%)	0.078
IA2 title of patients with positive IA2 (U/mL), median (IQR)	10.5 (31.1)	4.1 (10.8)	0.093
Positive IAA, n/total (%)	4/38 (10.5%)	1/35 (2.9%)	0.359

* Independent-samples Mann–Whitney test

** Chi square test

*** Independent-samples T-test

The proportion of female patients in both groups was similar. The median age at diabetes diagnosis was 38 years for T1DM (range 30–65 years, IQR 15) and 42 years for LADA (range 30–76, IQR 15). The median time between diabetes diagnosis and starting insulin therapy (which was the discriminative criteria of patients in the two groups) was 0 months in T1DM (range 0–4 months, IQR 0.0) and 48 months in LADA (range 6–396 months, IQR 56.3).

The recognition of the autoimmune aetiology for diabetes was defined as the time of measurement of diabetes-related autoantibodies. The median interval time between both diagnoses was 0 months in T1DM (range 0–216 months, IQR 3.0) and 60 months in LADA (range 0–444 months, IQR 92.3), *p* < 0.001. Included patients were followed for a median of 8 years for T1DM group (range 0.1–29.0 years, IQR 7.5) and 11 years for LADA group (range 1.0–43.0 years, IQR 10.5).

At the diagnosis of diabetes, 87% of T1DM patients and 45.7% of LADA patients presented with symptoms (*p* < 0.001). Among these, two patients with T1DM presented with diabetic ketoacidosis, and all the remaining patients presented with polyuric/polydipsic syndrome.

At the diagnosis of the autoimmune aetiology, T1DM group had a mean BMI in the normal range (mean 24.1 ± 3.8 kg/m^2^) and lower than the mean BMI in the LADA group, which was in the range of overweight (mean 26.1 ± 5.2 kg/m^2^); the latter difference was statistically significant (*p *= 0.042). At this point, T1DM had numerically (but not statistically) higher A1C and lower C-peptide.

In what concerns to the number of positive diabetes-related autoantibodies, T1DM patients had a median of 2 positive antibodies and LADA patients had a median of 1 positive antibody (*p *= 0.013). In T1DM group, 67.4% of the patients had more than one positive autoantibody, comparing to 41.3% of LADA patients (*p *= 0.012). GADA was the most prevalent positive autoantibody in both groups. The number of patients with positive GADA, ICA, IA2 and IAA was numerically higher in T1DM group comparing to LADA group and the titles of GADA and IA2 were higher in T1DM (not statistically significant).

In what concerns to the therapeutic regimen on the last follow-up (Table 2), all the patients in T1DM group were treated with insulin. Four patients in LADA group remained insulin-free. These patients had a diabetes duration from 4 to 10 years, were all under metformin and dipeptidyl peptidase-4 inhibitors (DPP4-i) and one was also under a sulfonylurea. At the last visit, their A1C values were 6.4%, 7.3%, 7.5% and 8.3%.

**Table 2:** Therapeutics on last follow-up

Insulin	T1DM (n = 44)	LADA (n = 46)	*p*
No insulin, n (%)	0 (0%)	4 (8.7%)	0.117
Basal insulin, n (%)	3 (6.8%)	5 (10.9%)	0.714
Premixed insulin, n (%)	6 (13.6%)	5 (10.9%)	0.689
Basal-plus, n (%)	1 (2.3%)	4 (8.7%)	0.361
Basal-bolus (MDI), n (%)	32 (72.7%)	27 (58.6%)	0.161
CSII, n (%)	2 (4.6%)	1 (2.2%)	0.612
Total daily dose (IU), median (IQR)	40.0 (32.0)	33.5 (33.0)	0.819
Total daily dose (IU)/Weight (kg), mean (± SD)	0.58 (± 0.31)	0.57 (± 0.39)	0.887
Non-insulin drugs			
Total, n (%)	9 (20.5%)	25 (54.3%)	0.001*
Metformin, n (%)	7 (15.9%)	22 (47.8%)	0.001*
Sulfonylureas, n (%)	0 (0%)	1 (2.2%)	1.000
DPP4-i, n (%)	5 (11.4%)	17 (37.0%)	0.005*
GLP1-ra, n (%)	0 (0%)	1 (2.2%)	1.000
SGLT2-i, n (%)	0 (0%)	2 (4.3%)	0.495

MDI: multiple daily injections; CSII: continuous subcutaneous insulin infusion

* Chi square test

A higher proportion of patients with T1DM were under basal-bolus insulin therapy: 72.7% were under basal-bolus therapy with multiple daily injections (MDI) comparing to 58.6% on LADA group; 2 patients with T1DM and 1 patient with LADA were under continuous subcutaneous insulin infusion (CSII). Remarkably, three T1DM patients were under basal insulin only; these patients had a short duration of disease (between 1 and 2 years), and A1C on last follow-up was 5.5%, 5.7% and 7.4%.

The median total insulin daily dose (TDD) was 40.0 IU in T1DM group and 33.5 IU in LADA group, which corresponded to 0.58 IU/kg in T1DM and 0.57 IU/kg in LADA. There were no statistically significant differences in TDD, before and after adjustment for body weight.

Non insulin drugs were more often used in LADA patients: 54.3% comparing to 20.5% in T1DM (*p *= 0.001). Metformin was prescribed to 47.8% of LADA patients and 15.9% of T1DM patients (*p *= 0.001); DPP4-i were prescribed to 37% of LADA patients, comparing to 11.4% of T1DM patients (*p *= 0.005). No T1DM patient was under sulfonylureas, glucagon-like peptide-1 receptor agonists (GLP1-ra) or sodium-glucose co-transporter-2 inhibitors (SGLT2i), which were prescribed in a minority of LADA patients.

Table 3 summarizes data concerning autoimmune (AI) comorbidities. The majority of patients in each group had no concomitant AI diseases: 68.9% in T1DM group and 65.2% in LADA group. T1DM group had more often multiple AI diseases, with 5 patients having more than one condition comparing to 2 patients in LADA group (not statistically significant). The most prevalent condition was AI thyroiditis in both groups, followed by atrophic gastritis, Graves’ disease (only present in the LADA group) and vitiligo. None of the patients had the diagnosis of celiac disease.

**Table 3:** Autoimmune diseases

	T1DM (n = 45)	LADA (n = 46)	P
Patients with no AI disease, n (%)	31 (68.9%)	30 (65.2%)	0.710
Patients with 1 AI disease, n (%)	9 (20.0%)	14 (30.4%)	0.252
Patients with > 1 AI diseases, n (%)	5 (11.1%)	2 (4.3%)	0.267
Organ-specific autoimmune diseases			
Autoimmune thyroiditis, n (%)	12 (26.7%)	8 (17.4%)	0.285
Graves disease, n (%)	0 (0.0%)	4 (8.7%)	0.117
Atrophic gastritis, n (%)	6 (13.3%)	2 (4.3%)	0.158
Vitiligo, n (%)	1 (2.3%)	2 (4.3%)	1.000
Addison disease, n (%)	1 (2.3%)	0 (0.0%)	0.495
Lupus, n (%)	0 (0.0%)	1 (2.2%)	1.000
Autoimmune hepatitis, n (%)	1 (2.3%)	0 (0.0%)	0.495
Sjogren, n (%)	0 (0.0%)	1 (2.2%)	1.000

The percentage of females with AI comorbidities was 66.7%, versus 44.3% without (*p *= 0.044); there were no gender differences between T1DM and LADA. Female patients with AI thyroiditis corresponded to 70.0%, comparing to 46.5% without this condition (*p *= 0.063, not significant). When we consider T1DM and LADA separately, the percentage of males with AI thyroiditis was 41.7% and 12.5%, respectively (*p *= 0.187, not significant).

Data regarding diabetic complications at 10 years of diabetes evolution is showed in Table 4. LADA patients had a statistically significant higher prevalence of microvascular complications: 63.3% comparing to 21.1% of T1DM patients (*p *= 0.004). This occurred mainly due to diabetic nephropathy, which was present in 53.3% of LADA and 23.5% of T1DM (*p *= 0.047). LADA group had a numerically higher proportion of patients with GFR lower than 90 or 60 mL/min, as well as Albuminuria A3 (not statistically significant). Three patients in LADA group had retinopathy (mild non proliferative diabetic retinopathy in all cases), while no T1DM patient had this complication. LADA also had more often peripheral neuropathy (3 patients). T1DM patients had a non-statistically significant trend to a higher prevalence of macrovascular complications.

**Table 4:** Diabetic complications at 10 years of diabetes duration

	T1DM	LADA	*P*
Microvascular complications, n/total (%)	4/19 (21.1%)	19/30 (63.3%)	0.004*
Peripheral neuropathy, n/total (%)	0/19 (0.0%)	3/30 (10.0%)	0.273
Retinopathy, n/total (%)	0/18 (0.0%)	3/26 (11.5%)	0.258
Nephropathy, n/total (%)	4/17 (23.5%)	16/30 (53.3%)	0.047*
GFR (mL/min), median (IQR) [n]	98.0 (19.8) [16]	93.0 (23.5) [28]	0.102
GFR < 90 mL/min, n/total (%)	4/16 (25.0%)	13/28 (46.4%)	0.160
GFR < 60 mL/min, n/total (%)	2/16 (12.5%)	8/28 (28.6%)	0.283
Albumin/creatinine ratio (mg/g), median (IQR) [n]	3.8 (12.4) [15]	8.7 (16.6) [26]	0.149
Albuminuria A3, n/total (%)	0/15 (0.0%)	3/26 (11.5%)	0.287
Macrovascular complications, n/total (%)	4/20 (20.0%)	1/31 (3.2%)	0.071
Ischemic Heart Disease, n/total (%)	1/19 (5.3%)	1/31 (3.2%)	1.000
Cerebrovascular disease, n/total (%)	3/19 (15.8%)	0/30 (0.0%)	0.053
Peripheral artery disease, n/total (%)	1/20 (5.0%)	0/31 (0.0%)	0.392

* Chi square test

Clinical and biochemical characteristics of patients at the last evaluation are presented in Table 5. There were no statistically significant differences between both groups in what concerns to A1C, although LADA group showed a trend to higher values. 71.7% of T1DM patients and 76.1% of LADA patients had non-optimized metabolic control (A1C over 7%). LADA group had a numerically higher BMI than T1DM, as well as a higher proportion of overweight and obese patients (not significant). LADA group also had a non-statistically significant higher proportion of patients with hypertension and dyslipidaemia. In what concerns to MetS, 82.6% of LADA patients and 55.6% of T1DM patients fulfilled the diagnostic criteria, with a statistically significant difference (*p *= 0.005). This difference remained statistically significant after adjustment for diabetes duration (OR = 1.10, 95% CI 1.02–1.19, *p *= 0.014).

**Table 5:** Clinical and biochemical characteristics at last follow-up

	T1DM	LADA	*p*
Diabetes duration at the last follow-up, years, median (IQR)	8.0 (7.5)	11.0 (10.5)	0.023*
A1C (%), median (IQR)	7.7 (1.9)	8.2 (1.7)	0.268
BMI (kg/m^2^), mean (± SD) [n]	25.6 (± 4.3) [42]	27.4 (± 5.2) [41]	0.086
Normal weight, n/total (%)	21/42 (50.0%)	13/41 (31.8%)	0.090
Overweight, n/total (%)	14/42 (33.3%)	14/41 (34.1%)	0.938
Obesity, n/total (%)	7/42 (16.7%)	14/41 (34.1%)	0.067
Hypertension, n/total (%)	25/46 (54.3%)	30/46 (65.2%)	0.288
Dyslipidaemia, n/total (%)	29/46 (63.0%)	35/46 (76.1%)	0.174
Total cholesterol, mean (± SD)	183.7 (± 41.4)	177.6 (± 41.6)	0.494
HDL cholesterol, median (IQR)	53.0 (26.0)	46.3 (14.0)	0.128
LDL cholesterol, mean (± SD)	113.6 (± 39.4)	113.6 (± 37.3)	0.998
Triglycerides, median (IQR)	85.0 (43.0)	93.5 (95.0)	0.371
Metabolic syndrome, n/total (%)	25/45 (55.6%)	38/46 (82.6%)	0.005**

*Independent-samples Mann–Whitney test

**Chi square test

## Discussion

### Baseline characteristics

AID is a very heterogeneous disease in what concerns to the pathophysiological mechanisms (genetic background, autoimmune process, environmental factors), which leads to a spectrum of clinical profiles with variable degrees of insulin deficiency and insulin resistance [1, 5, 9]. The 2005 IDS diagnostic criteria of LADA [4], although highly applied in clinical practice, raise many questions, such as the lower limit for age (excluding latent autoimmune diabetes of the young [10]) and the subjectivity of the onset of insulin therapy, which is dependent on the physician’s decision [1, 4]. Other scientific societies propose different designations and diagnostic criteria for this clinical entity; this is the case of the Japan Diabetes Society, that considers “Slowly progressive insulin-dependent diabetes mellitus (SPIDDM)”, whose diagnostic criteria are (1) the presence of GADA and/or ICA at some time during the disease course and (2) absence of ketosis at onset of DM and no need for insulin treatment to correct hyperglycaemia in the first 3 months after diagnosis [11]. The World Health Organization Classification of Diabetes Mellitus 2019 considers “Slowly evolving, immune mediated diabetes of adults” as a hybrid form of diabetes, although no definitive diagnostic criteria are proposed due to the controversies regarding classification as a separate subtype of diabetes or as a stage of T1DM [12]. Considering their frequent use and the fact that they are easy to apply in clinical practice, we decided to use the IDS criteria in our study. Therefore, we aimed to characterize adult patients (over 30 years old) with AID, comparing patients with classic T1DM and LADA.

In our sample, T1DM patients presented more often with symptoms at diagnosis, which may have justified the early institution of insulin therapy. At the diagnosis, patients with AID may present variable clinical phenotypes, ranging from ketoacidosis to asymptomatic hyperglycaemia that can be controlled with diet alone [5]. In this study, 6 patients in T1DM group were asymptomatic and 21 patients in the LADA group had polyuric/polydipsic syndrome. Notably, only 2 patients in T1DM group presented with diabetic ketoacidosis, which did not occur in any patient in the LADA group.

The recognition of the autoimmune aetiology of diabetes, defined as the time of measurement of autoantibodies, occurred at the same moment of the diagnosis of diabetes for most patients on T1DM group. Nevertheless, in 5 patients within this group, the measurement of autoantibodies was performed years after diabetes diagnosis (from 3 to 18 years), although these patients were always under insulin therapy. On the other hand, in the LADA group there was a large interval between the onset of diabetes and the establishment of the autoimmune aetiology: median of 5 years (60 months), with a maximum of 37 years. This may be due to the fact that LADA patients are often misdiagnosed as having T2DM [1, 5, 13]. A multicentric Spanish study reported a delay of 3.5 years in LADA confirmation [13], which was similar to our study.

At the diagnosis of the autoimmune aetiology, the T1DM group had a statistically significant lower BMI comparing to LADA, which is supported by literature [9, 14, 15]. This difference may be explained by the important role of lifestyle, leading to insulin resistance in LADA patients [6], as we discuss later. At this same time point, T1DM group had a trend to higher A1C and lower C-peptide, with no statistically significant difference between groups. Other studies reported significant differences in A1C and C-peptide, being respectively higher and lower in T1DM comparing to LADA [9, 14, 15]. This may be justified by the hypothesis of a more aggressive autoimmune process, with more severe insulinopenia in T1DM patients [1, 2].

### Autoimmunity

In what concerns to diabetes-related autoantibodies, T1DM patients had more often multiple positive antibodies and had higher titles of GADA, which has been reported in other studies [2, 5, 15, 16]. It has also been reported that among LADA patients, those with higher number of positive antibodies and higher titles of GADA have a “T1DM-like” phenotype, comparing to those with only one positive antibody and lower GADA titles who have a “T2DM-like” phenotype [1, 2, 5, 17]. However, other studies suggest that the presence of GADA, independently of the title, highly increases the risk of progression to insulin dependence, comparing to T2DM [18].

Autoantibodies do not seem to be the key pathogenic factor in AID, but rather a marker of a process that appears to be mediated by immune cell response [1]. LADA patients share genetic variants in human leukocyte antigen (HLA) complex with T1DM patients, which confer susceptibility to AID [16, 19, 20]. Nevertheless, in T1DM patients the autoimmune process is more aggressive, leading to severe beta-cell destruction, insulinopenia and risk of ketosis [1, 6]. T2DM-risk genetic variants are not so common in LADA patients [19, 20] and the pathological mechanisms of beta-cell failure seem different between LADA and T2DM [16]. However, these two populations seem to share the unhealthy lifestyle, which increases the risk of overweight, increased adiposity and insulin resistance [6, 20]. In fact, in our sample LADA patients had more often MetS comparing to T1DM patients. Therefore, in LADA both insulin deficiency and resistance play important roles in the pathogenesis [5, 6, 9].

In our sample, more than two-thirds of patients in both groups did not have other autoimmune diseases. Nevertheless, the few T1DM who had other autoimmune disorders had more often multiple conditions, which can be a sign of a more aggressive autoimmune process [1, 5, 6]. The most frequent comorbidity was thyroid autoimmune disorder. Interestingly, no patient had the diagnosis of celiac disease, which is frequently associated to childhood T1DM [21].

In this study, female patients had more often other AI conditions, namely AI thyroiditis. Zampetti et al. [17] reported a higher prevalence of thyroid antibodies in male LADA patients with higher titles of GADA. In our sample, the percentage of male patients with AI thyroiditis was superior in T1DM group, reinforcing the concept that a more intense autoimmune profile increases the risk of AI thyroiditis in male patients.

### Diabetes treatment

In what concerns to diabetes treatment at the last evaluation, in our sample all T1DM patients were under insulin therapy, mainly basal-bolus regimen. Most LADA patients were under basal-bolus therapy: a total of 58.6%, which seems lower than other series [13]. The mean TDD adjusted for weight was very similar between both groups (0.58 IU/kg in T1DM and 0.57 IU/kg in LADA).

Although LADA has distinct pathophysiological mechanisms of disease comparing to T1DM (less pronounced insulinopenia and significant insulin resistance), with the progression of the disease most patients eventually need insulin therapy [1, 3, 13]. Most studies on LADA compare these patients with T2DM and describe a faster progression to insulin therapy, mainly in patients with higher GADA titles [1, 2, 5]. Nevertheless, in our sample four LADA patients (8.7%) were not under insulin treatment, with no episodes of ketosis (three of them with good glycaemic control). On the other hand, 54.3% of LADA patients were under non-insulin antidiabetics (comparing to 20.5% of T1DM group), which seems lower than other series [13, 22].

### Metabolic control and diabetes complications

In the field of diabetes complications, at 10 years of diabetes duration, we report a significant higher frequency of microvascular complications in LADA, especially due to diabetic nephropathy. The slow progression of disease in LADA may be associated with asymptomatic hyperglycaemia before diagnosis, which together with other metabolic risk factors, leads to a continuous micro and macrovascular damage [1, 15]. There are reports in the literature of LADA patients with established micro and macrovascular complications in the first year after diabetes diagnosis [15]. In our study, patients in the LADA group showed a trend to have higher weight, HbA1c and blood pressure values, and this may have contributed to the increased frequency of nephropathy in this group.

There are not many studies comparing long term diabetes complications between T1DM and LADA. For identical disease duration, the frequency of albuminuria and chronic kidney disease seems to be identical between LADA and T2DM, being higher in these groups comparing to T1DM [22]. However, a post hoc analysis from UKPDS study showed a lower risk of microvascular complications at diabetes onset in LADA patients comparing to T2DM, followed by a higher risk after 9 years of disease due to worse glycaemic control [23]. In what concerns to cardiovascular disease, it seems to occur at a higher frequency in T2DM, comparing to LADA and T1DM [22, 24]. Our study showed a non-statistically significant trend to a higher proportion of T1DM patients with macrovascular complications. Literature data regarding differences between LADA and T1DM in this field are conflicting: Luk et al. report similar long-term frequencies in cardiovascular outcomes [22], while Wod et al. report a lower prevalence in LADA [24].

In what concerns to glycaemic control on last follow-up, LADA patients had a non-statistically significant higher A1C (8.2% comparing to 7.7% in T1DM) and a higher percentage of patients with A1C over 7%. A possible bias is the longest median duration of disease in LADA group (11 years versus 8 years in T1DM). Nevertheless, Luk et al. reported identical mean A1C values for T1DM and LADA (8.5% and 8.4%), after a median duration of diabetes of 8 and 6 years respectively [22]. On the other hand, LADA group had a statistically significant higher proportion of patients with MetS: 82.6% comparing to 55.6% of T1DM patients. This difference between groups remained significant after adjustment for diabetes duration. The proportion of MetS in LADA is comparable to its prevalence in T2DM patients [25]. When we consider isolated components of MetS, LADA patients had a non-statistically significant trend to have more often BMI over 25 kg/m^2^, hypertension and dyslipidaemia, which confirms other published results [21]. These differences reflect the important role of adiposity and insulin resistance in the pathogenesis of LADA and its related complications [1, 3, 6].

### Strengths and limitations on this study

This is a retrospective study with the limitations related to this kind of design: the unavailability of all data in patients’ files and the non-uniform diagnostic and therapeutic strategies. The non-matching of patients in what concerns to disease duration in the groups may lead to some difficulties in interpreting the results. Nonetheless, the patients included in the study represent a “real-world” sample of diabetic patients followed on a tertiary care centre. To the best of our knowledge, few studies compared T1DM and LADA patients, especially with long duration of disease.

## Conclusions

Our study showed that AID is a very heterogeneous condition, with a clinical presentation that may range from asymptomatic hyperglycaemia to diabetic ketoacidosis. Patients with classic T1DM presented more often with symptoms at diagnosis, lower BMI and higher number of autoantibodies, which may be related to a more aggressive autoimmune process. This symptomatic presentation is probably the main factor leading to an early institution of insulin therapy. Patients with LADA developed more frequently microvascular complications and particularly nephropathy, also having a trend to a higher prevalence of peripheral neuropathy and retinopathy. This may be related with a more insidious nature of the disease, with higher glucose exposure, as well as with higher levels of insulin resistance and its associated comorbidities. LADA patients also had a higher prevalence of metabolic syndrome, which strengths the role of adiposity and insulin resistance in the pathophysiology of this type of diabetes and its complications.

## Data Availability

The datasets used and/or analysed during the current study are partially included within the article. Complete datasets are available from the corresponding author on reasonable request.

## Abbreviations

A1C: Haemoglobin A1c
AI: Autoimmune
AID: Adult-onset autoimmune diabetes
BMI: Body mass index
C-pep: C-peptide
CSII: Continuous subcutaneous insulin infusion
DPP4-i: Dipeptidyl peptidase-4 inhibitors
DM: Diabetes mellitus
GADA: Glutamic acid decarboxylase antibodies
GFR: Glomerular filtration rate
GLP1-ra: Glucagon-like peptide-1 receptor agonists
HDL-c: High-density lipoprotein cholesterol
HLA: Human leukocyte antigen
IAA: Insulin autoantibodies
IA2: Tyrosine phosphatase-like insulinoma antigen 2
ICA: Islet cell antibodies
IDS: Immunology of Diabetes Society
LADA: Latent autoimmune diabetes in adults
LDL-c: Low-density lipoprotein cholesterol
MDI: Multiple daily injections
MetS: Metabolic syndrome
SGLT2i: Sodium-glucose co-transporter-2 inhibitors
T1DM: Classic type 1 diabetes mellitus
T2DM: Type 2 diabetes mellitus
TDD: Total insulin daily dose
